# *In Silico* Identification of QTL-Based Polymorphic Genes as Salt-Responsive Potential Candidates through Mapping with Two Reference Genomes in Rice

**DOI:** 10.3390/plants9020233

**Published:** 2020-02-11

**Authors:** Buddini Abhayawickrama, Dikkumburage Gimhani, Nisha Kottearachchi, Venura Herath, Dileepa Liyanage, Prasad Senadheera

**Affiliations:** 1 Department of Biotechnology, Faculty of Agriculture and Plantation Management, Wayamba University of Sri Lanka, Makandura, Gonawila 60170, Sri Lanka; bpramudika@wyb.ac.lk (B.A.); dgimhani@wyb.ac.lk (D.G.); dileepasripal@gmail.com (D.L.); 2 Department of Agricultural Biology, Faculty of Agriculture, University of Peradeniya, Peradeniya 20400, Sri Lanka; venura@agri.pdn.ac.lk; 3 Department of Botany, Faculty of Natural Sciences, Open University of Sri Lanka, Nawala 11222, Sri Lanka; spsen@ou.ac.lk

**Keywords:** abiotic stress, rice, salinity, whole genome re-sequencing

## Abstract

Recent advances in next generation sequencing have created opportunities to directly identify genetic loci and candidate genes for abiotic stress responses in plants. With the objective of identifying candidate genes within the previously identified QTL-hotspots, the whole genomes of two divergent cultivars for salt responses, namely At 354 and Bg 352, were re-sequenced using Illumina Hiseq 2500 100PE platform and mapped to Nipponbare and R498 genomes. The sequencing results revealed approximately 2.4 million SNPs and 0.2 million InDels with reference to Nipponbare while 1.3 million and 0.07 million with reference to R498 in two parents. In total, 32,914 genes were reported across all rice chromosomes of this study. Gene mining within QTL hotspots revealed 1236 genes, out of which 106 genes were related to abiotic stress. In addition, 27 abiotic stress-related genes were identified in non-QTL regions. Altogether, 32 genes were identified as potential genes containing polymorphic non-synonymous SNPs or InDels between two parents. Out of 10 genes detected with InDels, tolerant haplotypes of *Os01g0581400*, *Os10g0107000*, *Os11g0655900*, *Os12g0622500,* and *Os12g0624200* were found in the known salinity tolerant donor varieties. Our findings on different haplotypes would be useful in developing resilient rice varieties for abiotic stress by haplotype-based breeding studies.

## 1. Introduction

Rice, being the staple food crop of many nations, is considered as a high priority crop for research programs that focused on ensuring food security [1,2,3]. Rice is mostly cultivated under natural rain-fed systems frequently exposed to various abiotic and biotic stress conditions throughout the world. Development of improved rice varieties for abiotic stress tolerance is the most affordable strategy to increase rice production using marginal and non-arable lands. Among major abiotic stress conditions, salinity, the presence of increased levels of salts, predominantly sodium chloride, is considered the second most limiting factor for rice production next to drought [4]. In every year, nearly two million hectares of irrigated land become uncultivable due to the buildup of salts [5]. In addition, sodic soil which is accumulated with excessive sodium ions cause unfavorable conditions for agriculture by adversely affecting the soil physical properties. Thus, the interaction between soil sodicity and salinity could seriously compromise the rice growth in the field [6,7]. However, due to the genetic complexity of the trait, development of resilient varieties against salinity stress cannot be achieved by a single step strategy. Due to the polygenic nature of the trait, many Quantitative Trait Loci (QTLs) and Quantitative Trait Nucleotides (QTNs) have been reported linking either with salinity tolerance or susceptibility traits distributed throughout the genome in many rice lines [8,9,10,11,12,13].

Although rice is sensitive to salt, especially at the seedling stage and reproductive stage, vast diversity for this trait across the rice varieties offers a promising tool for improving salt tolerance in rice. Pokkali and Nona bokra are popular traditional salt-tolerant *indica* rice varieties that tolerate up to 80 mM NaCl at the seedling stage and serve as donors for rice salt tolerance [14]. The major strategies for improving salinity tolerance are reducing Na^+^ toxicity by limited Na^+^ net influx, Na^+^ compartmentalization and removal of Na^+^ into the apoplast to achieve a good Na^+^/K^+^ balance in the shoot under saline condition [3]. It is reported that Pokkali, demonstrates both ‘Na^+^ exclusion’ and ‘ion balance’ mechanisms while Nipponbare, a moderate tolerant *japonica* variety showed only ‘ion balance’ [14]. Besides, accumulation of compatible osmolytes for osmotic protection, antioxidant regulation and minimalizing the exposure time of cells to ionic imbalance are observed as components of salt tolerance [15,16,17]. By QTL mapping, genomic locations of such mechanisms are primarily recognized, giving an insight into the understanding of gene-level identification. Fine mapping followed by map-based cloning is the common approach that has been practicing to reveal candidate genes from QTLs [18,19]. For example, *SKC1* gene that encodes HKT-type transporter is one of the salinity tolerant genes identified through dissecting *Saltol* QTL by map-based cloning [20]. Harnessing QTLs and QTNs of salinity tolerance from diverse rice accessions and introgression them to generate salt-tolerant varieties can be achieved by marker-assisted breeding, which is based on genomic sequences. The Next Generation Sequencing (NGS) technique has been successful in generating DNA sequences of organisms revealing genomic variations at a low cost. It is becoming more popular than the use of marker-based polymorphism techniques. There are many studies indicating how NGS facilitates rice improvement by exploration and exploitation of many functional genes that regulate agronomic traits [21,22,23,24].

Feltus et al. (2004) [25] have reported that there are 408,898 candidate DNA polymorphisms including single nucleotide polymorphisms (SNPs) and InDels distinguished between *indica* and *japonica*. These SNPs and InDels can be exploited for gene mapping, association studies and DNA marker-assisted breeding. If there is an SNP or InDel in a gene or regulatory sequence of the gene, there will be a chance to affect the function of the gene either adversely or favorably relative to the function of the gene of reference genome by creating either a missense mutation or premature termination or preventing stop signal or shifting the amino acid sequence leading to phenotypic variations. Mishra et al. (2016) [26] have reported that some of SNP haplotypes of HKT family genes were associated with salt tolerance in Indian wild rice germplasms while some other SNP haplotypes were sensitive to salt stress indicating the impact of SNP variations for the phenotype. The popular *SUB1A* allele of ethylene response factor-like gene that carries an SNP mutation conferring submergence tolerance in vegetative stage of rice is another evidence for the contribution of SNP mutation towards favorable agronomic traits [27]. Therefore, mining of SNPs and InDels of candidate genes is useful for detecting possible phenotypic variations which would be important in breeding programs.

The availability of whole genome information, gene expression profiles and *in silico* gene annotation tools have enabled physical identification of candidate genes by aligning genetic map and the putative QTLs. This approach helps to shortlist promising candidate genes of the trait by analyzing SNPs, InDels and structural variations which can later be validated by expression studies and promoter analysis. Instead of costly conventional fine mapping done with large inbred populations that need significant labor and time, QTLs and QTNs targeted annotation of the NGS derived sequences has revealed many candidate genes in various disciplines of plants [2,8,10,28,29].

Many researchers have conducted QTL mapping studies using Simple Sequence Repeat (SSR) markers, but they could not develop genetic linkage maps with more than 300 markers due to lack of polymorphism [30,31]. Therefore, SSR marker-based maps usually generate many gaps that are difficult to be used directly in candidate gene discovery studies. Currently, SNP markers have become more popular as they generate the vast number of polymorphic sites among individuals. For example, Thomson et al. (2017) [32] have reported that usually 1300–2500 SNP polymorphic markers could be generated from a bi-parental population of rice derived from either *indica* × *indica* or *indica* × *japonica*, if Cornell_6K_array_Infinium_Rice (C6AIR) chip containing about 6000 SNPs is used. Therefore, it appears that due to the availability of huge re-sequencing data, high-density SNPs-based maps have been developed [33,34]. Gimhani et al. (2016) [35] were also able to produce SNP-based highly dense and saturated molecular maps with the C6AIR chip covering 1460.81 cM of the rice genome with an average interval of 1.29 cM between marker loci using a Recombinant Inbred Line (RIL) population derived from At 354 and Bg 352. At 354 is a salinity tolerant elite rice *indica* variety with the pedigree of Pokkali and Bg 94-1 and Bg 352 is a salinity susceptible elite rice *indica* variety with the pedigree of Bg 380/Bg 367-4. Both At 354 and Bg 352 are recommended, high yielding, improved rice varieties in Sri Lanka with a relatively short growth duration of 105 days. Gimhani et al. (2016) reported 14 QTL hotspots and 11 solitary QTLs for salt tolerance flanked with SNP markers narrowing down to less than 1 Mb intervals indicating the potential of use in gene mining studies. We noted that the same regions of these QTLs were reported in other studies validating the potentiality of accommodating candidate genes for abiotic stress, mapped using other breeding populations (Supplementary Table S1). Therefore, it is worthwhile for attempting physical identification of the particular regions via NGS-based approaches instead of conventional fine mapping techniques that consume much time. Hence, as an extension of the same study, we sequenced two varieties—At 354 and Bg 352—with reference to *Oryza sativa japonica* group cultivar Nipponbare and *Oryza sativa indica* group cultivar Shuhui498 (R498) and reported revealing of candidate genes underlined by those QTL hotspots. We performed a gene ontology (GO) analysis to functionally characterize the potential candidate genes. We also outlined the variant calling procedure of the At 354 and Bg 352 genomes, the short-listing approach of the candidate genes leading to salinity tolerance and their possible allelic differences.

## 2. Results

### 2.1. Whole Genome Sequencing and Comparison with Nipponbare and R498 Reference Genomes

Whole genome sequencing of At 354 and Bg 352 generated 11.5 and 13.5 Gb of raw data, respectively. More than 90% of the data exceeded Q30 Phred quality score for both of the varieties with mean depth coverage of 30X. The GC percentages of At 354 and Bg 352 were found to be 42.75 and 49.03 respectively. The reads of At 354 and Bg 352 were aligned to two reference genomes. *Oryza sativa japonica* group cultivar Nipponbare IRGSP-1.0 with 374,304,577 bp length was used as the reference genome and the mapped lengths of At 354 and Bg 352 were 349,124,521 bp (93.27%) and 348,205,846 bp (93.03%) respectively. Out of total generated reads, 108 × 10^6^ reads of At 354 and 96 × 10^6^ reads of Bg 352 were aligned to the Nipponbare genome with an average of 27.9X and 24.9X read depth and 94.65% and 70.76% genome wide coverage respectively (Table 1). Also, the reads were aligned to *Oryza sativa indica* group cultivar R498 with a length of 390,322,188 bp and more than 95% of the length of R498 genome was mapped to At 354 (374,732,599 bp) and Bg 352 (373,811,968 bp). Out of total generated reads, 110 × 10^6^ reads of At 354 and 96 × 10^6^ reads of Bg 352 were aligned to the R498 genome with an average of 27.3X and 24.4X read depth and 96.98% and 72.15% genome wide coverage, respectively (Table 1).

### 2.2. Identification of Variants in At 354 and Bg 352 Genomes

**Figure 1:**
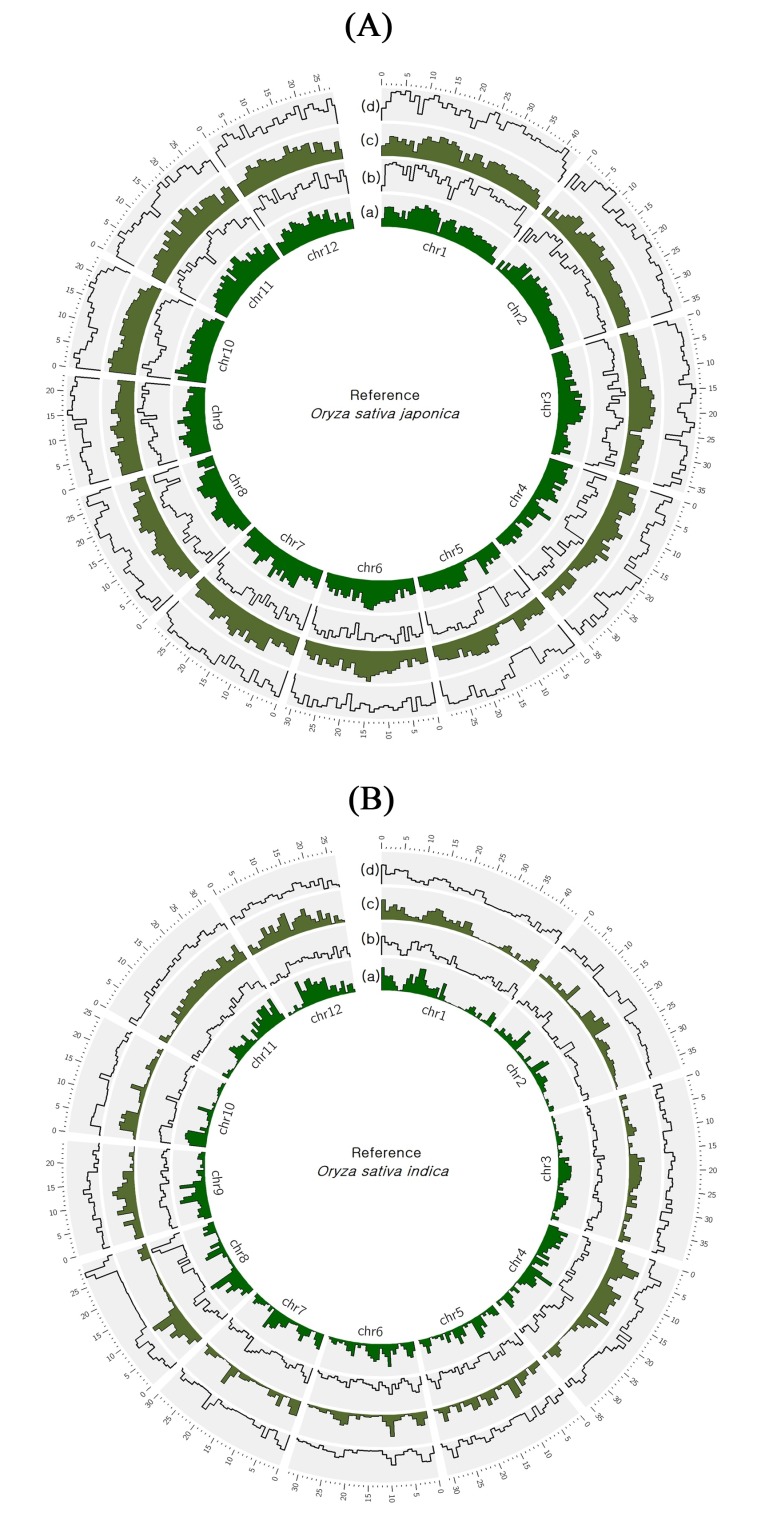
Frequency distribution of single nucleotide polymorphisms (SNPs) and InDels in At 354 and Bg 352. (**A**) with reference to Nipponbare, (**B**) with reference to R498. (a) SNPs–At 354 (b)–InDels At 354 (c) SNPs–Bg 352 (d) InDels–Bg 352.

The genome-wide SNPs and InDels on At 354 and Bg 352 were examined with reference to the Nipponbare and R498 genomes. The frequency distributions of total SNPs and InDels of two varieties with respect to Nipponbare and R498 were shown in Figure 1. Identification of variants with comparison to Nipponbare genome showed that a total of 2,734,000 variants (2,478,369 SNPs and 255,631 InDels) in At 354 and a total of 2,726,469 variants (2,477,244 SNPs and 249,225 InDels) in Bg 352. With reference to R498, only 1,122,726 (1,044,783 SNPs and 77,943 InDels) and 1,107,112 (1,038,244 SNPs and 68,868 InDels) of total variants were observed in At 354 and Bg 352 respectively. The highest SNPs density was observed in chromosome 10 (776.5 and 778.4 in At 354 and Bg 352 respectively) in both varieties while the lowest was on chromosome 4 (557.9) and 5 (535.5) in At 354 and Bg 352 respectively with reference to Nipponbare. However, with reference to R498, the highest SNPs density was observed in chromosome 12 (357.8) in At 354 and chromosome 4 (421.2) in Bg 352 while the lowest SNPs density was observed in chromosome 2 (191.5) in At 354 and chromosome 7 (164.4) in Bg 352 respectively. Most of the SNP changes observed were of transition type with a Ts/Tv ratio of 2.54 in both varieties with respect to Nipponbare reference and a Ts/Tv ratio of 2.48 with respect to R498 reference genome. With regards to InDel density, the highest was observed in chromosome 2 and 3 while the lowest was in chromosome 12 and 4 in At 354 and Bg 352 respectively with reference to Nipponbare. With reference to R498, the highest InDel density was shown in chromosome 8 of both varieties while the lowest was shown in chromosome 3 and 10 of At 354 and Bg 352 respectively (Table 2).

### 2.3. QTL-Based SNPs and InDels of Abiotic Stress-Related Genes

**Figure 2:**
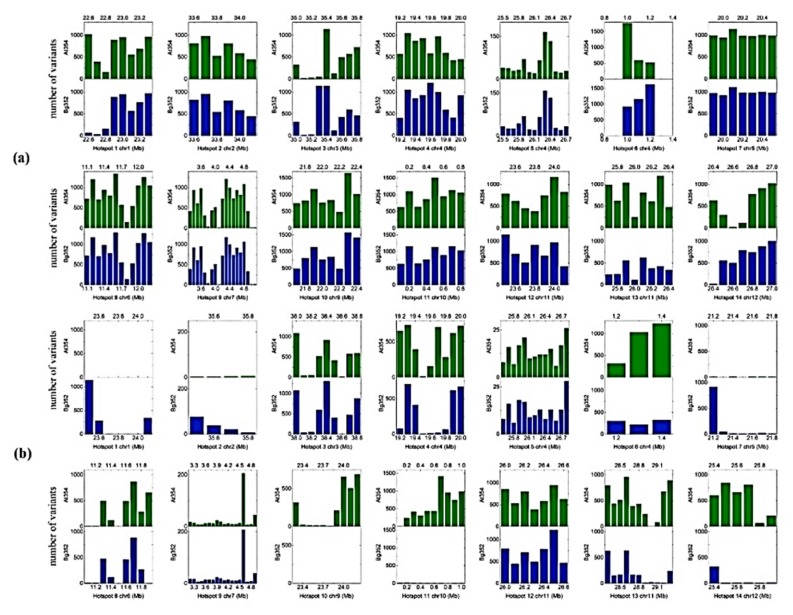
Location of 14 Quantitative Trait Loci (QTLs) on rice chromosomes and the total variants (SNPs and InDels) distribution within the QTLs in 100 kb windows. (**a**) with reference to Nipponbare, (**b**) with reference to R498.

QTL-based screening was performed on previously identified salinity stress-related QTL hotspots [35], and we observed slight deviations (0.1 Mb to 3.0 Mb) in the corresponding locations of QTL hotspots with reference to R498 (Figure 2). As expected, a low number of total variants were observed in R498 in each and every QTL examined compared to the Nipponbare. The most abundant variants were found in QTL hotspot 9 of At 354 parent with reference to Nipponbare while the least abundant variants were found in hotspot 10 of Bg 352 parent with reference to R498 (Supplementary Table S2). We found 1236 genes within QTL hotspots and the highest number of genes (215) was found on QTL hotspot 9 while the lowest number (51) was found on QTL hotspot 11. Out of them, 106 genes were associated with abiotic stress. The highest number of stress-related genes (19) was detected within QTL hotspot 2 located on chromosome 2. The lowest number of genes were on hotspots 6 and 12 located on chromosomes 4 and 11, respectively (Supplementary Table S2). In this study, we examined genes located in non-QTL regions to minimize the exclusion of other potential salinity-related genes. Accordingly, we selected 27 genes known for their association with salinity. Therefore, altogether 133 genes were used to examine the allelic differences for salinity.

In the above 133 genes, the variants located in exons, introns, 5′ UTR and 3′ UTR regions were analyzed (Supplementary Table S3). Accordingly, *Os01g0581400* which was reported as serine-threonine protein kinase-related domain-containing protein possessed 21 nucleotide variants in the 5′ UTR, 16 variants in the exons and 10 variants in the introns in At 354 comparatively to Nipponbare sequence while Bg 352 possessed only 1 nucleotide variant in 3′ UTR. *Os11g0661600* (similar to peroxidase), *Os11g0669100* (calmodulin binding protein-like family protein), *Os11g0621825* (similar to universal stress protein) were the genes with the highest number of nucleotide variants in exon regions in both of the varieties. *Os11g0621825* (a protein similar to universal stress protein) possessed 110 variants in At 354 and 129 variants in Bg 352 in the intron regions, comparatively to Nipponbare. *Os04g0423400* (similar to *OSIGBa0076I14.3* protein) possessed 10 and 11 nucleotide variants in At 354 and Bg 352 respectively in the 3’ UTR.

### 2.4. Screening Candidate Genes Based on Polymorphic Nucleotide Variants between Two Parents

This study was aiming at finding polymorphic nucleotide variants between two parents with the potential of salinity tolerance based on allelic differences. Therefore, we screened the genes that showed at least one difference in exons of the nucleotide sequence in one parent comparatively to the other parent from the above 133 gene list. As a result, we found 31 genes located within the QTL regions and three genes located outside the QTLs containing polymorphic variations in the exon region. Each one of them had either one of missense or frame shift or loss of stop codon or early gain of the start codon. Table 3 and Supplementary Table S4 shows the polymorphism type and the location of above 34 candidate genes extracted from the gene sequences of At 354 and Bg 352. Accordingly, we observed 84 variants including 72 SNPs and 12 InDels in 34 genes compared to Nipponbare reference while 73 variants including 63 SNPs and 10 InDels compared to R498 reference. Two InDel variants found in *Os01g0307500* and *Os04g0423400* with reference to Nipponbare were absent with reference to R498.

**Figure 3:**
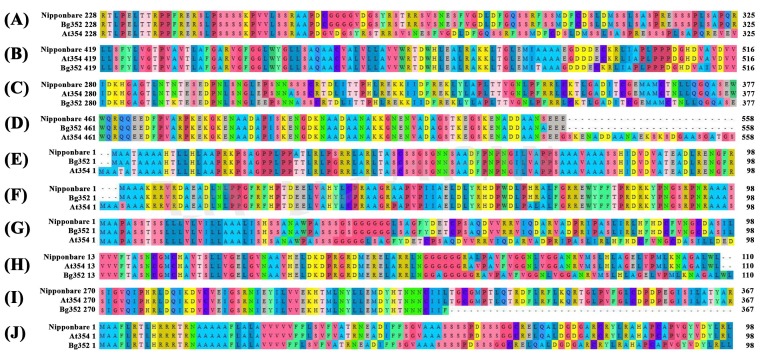
Sequence alignment showing the amino acid sequence coded by putative candidate genes detected with InDel variations. (**a**) *Os01g0581400*, (**b**) *Os03g0839200*, (**c**) *Os04g0117600*, (**d**) *Os05g0390500*, (**e**) *Os07g0181000*, (**f**) *Os07g0225300*, (**g**) *Os10g0107000*, (**h**) *Os11g0655900*, (**i**) *Os12g0622500*, (**j**) *Os12g0624200*.

*Os01g0581400* gene of At 354 (GenBank accession number: MK440689) was found with a 12 bp deletion and three missense mutations. In Bg 352, the gene (GenBank accession number: MK440690) encoded the full sequence with 765 amino acids while the sequence of At 354 shifted from 262 position and terminated with 761 amino acid residues due to the 12 bp deletion (Figure 3). GO analysis indicated that *Os01g0581400* was responsible for protein phosphorylation in relation to stress (Supplementary Table S5, [36]).

The gene *Os02g0766700* located within QTL hotspot 2 exhibited two missense mutations in Bg 352 leading to change in amino acid residues from lysine to asparagine and phenylalanine to leucine. According to GO analysis, this gene was reported to provide a regulatory function as a transcription factor in Abscisic Acid (ABA) signaling, water deprivation and salt stress (Supplementary Table S5, [37,38]. Another gene, *Os02g0782500*, located on the same QTL was found with one missense variant in At 354 which changed the glycine to serine.

The gene *Os03g0839200* on QTL hotspot 3 associated with protein detoxification had a 3 bp deletion and a 3 bp insertion at two different locations in Bg 352 (GenBank accession number: MK440692). These two mutations caused a change in amino acid sequence from 490 position in Bg 352 and terminated with 516 amino acids. In At 354, the gene (GenBank accession number: MK440691) indicated encoding the full sequence as of Nipponbare with 516 amino acids (Figure 3). Another gene *Os03g0795900*, a heat stress transcription factor associated with tolerance to environmental stress [39], was found with two missense variants in At 354 sequence, changing serine into alanine and proline into serine.

In Bg 352, the gene *Os04g0117600* (GenBank accession number: MK492739) had a 3 bp insertion and caused a frame shift in amino acid sequence starting from 310 position and terminated with 690 amino acids while At 354 (GenBank accession number: MK492738) showed encoding of full sequence with 689 amino acids. The gene is indicated as tRNA-dihydrouridine synthase-like gene [40]. Go analysis indicated that it could be involved in oxidation reduction biological processes.

*Os05g0390500* of At 354 (GenBank accession number: MK492742) exhibited 2 bp insertion which leads to the loss of stop codon and extended the sequence up to 537 amino acid residues. The Bg 352 (GenBank accession number: MK492743) sequence of the same gene indicated encoding for 536 amino acid protein similar to Nipponbare (Figure 3). This gene was located at QTL hotspot 7 from which the salt tolerance was contributed by At 354 parent as indicated by the additive effect of the QTL (Table 3). Although *Os06g0318500* was found with four missense alternative variants in Bg 352 with reference to Nipponbare, the gene was found only with three missense alternative variants with reference to R498, encoding three different amino acid residues in respective positions. GO analysis revealed that this gene functions similar to Sodium/hydrogen exchanger as reported by Panahi et al. (2013) and Reguera et al. (2014) [41,42].

*Os07g0181000* which is associated with kinase activity and ion binding exhibited 6 bp insertion in At 354 (GenBank accession number: MK492744) which resulted in extended amino acid sequence with 580 amino acids while Bg 352 (GenBank accession number: MK492745) had the complete sequence coding for 578 amino acid protein. *Os07g0225300* of At 354 (GenBank accession number: MK492754) showed an 8 bp insertion along with two missense variants. The 8 bp insertion has occurred just before the starting codon thereby leading for gaining of a start codon at three residues before the Nipponbare reference sequence (Figure 3).

The QTL hotspot 11 on chromosome 10 was spotted with five candidate genes in which two of them had frame shifts. In At 354, *Os10g0107000* (GenBank accession number: MK492746) which is responsive to oxidative stress [43] possessed a 3 bp deletion, 3 bp insertion and a 9 bp deletion causing a frame shift in amino acid sequence starting from 28 position and terminated with 326 amino acids while Bg 352 (GenBank accession number: MK492747) encoded the full length of sequence with 329 amino acids. *Os11g0621825,* which codes for a protein similar to universal stress protein [44], was found with two missense mutations in Bg352. The gene *Os11g0655900* which is important for cell redox homeostasis and electron transportation had a 6 bp insertion in At 354 (GenBank accession number: MK492750) causing a frame shift in its amino acid sequence starting from 65 position and terminating at 110 position. Both Bg 352 (GenBank accession number: MK492751) and Nipponabre coded for amino acid sequences with 108 amino acid residues (Table 3).

Out of three candidate genes of QTL hotspot 14 located on chromosome 12, two genes had frame shifts in Bg 352. A 2 bp insertion in *Os12g0622500* of Bg 352 resulted in a 323 amino acid protein due to early gain of stop codon while At 354 had the full sequence coding for 487 amino acids. The gene *Os12g0624200* was found with a 3 bp deletion in Bg 352 and the mutation caused a frame shift starting from the 30 position and terminating at 586 position while At 354 encoded as that of the Nipponbare sequence with 587 amino acids. According to GO analysis, this gene encodes an integral membrane protein that involves transport activity (Supplementary Table S5). In addition, *Os01g0583100*, *Os01g0591000*, *Os02g0148100*, *Os03g0838400*, *Os03g0839000*, *Os03g0848400*, *Os04g0116600*, *Os04g0430800*, *Os05g0393800*, *Os05g0455500*, *Os09g0559800*, *Os10g0103800*, *Os10g0105400*, *Os10g0109600*, *Os11g0656000*, *Os11g669100* and *Os12g0623500* exhibited different missense variants leading to amino acid residue changes in one parent compared to the other parent (Supplementary Table S4).

### 2.5. Comparative Analysis of InDels in Predicted Candidate Genes with indica Rice Lines

**Figure 4:**
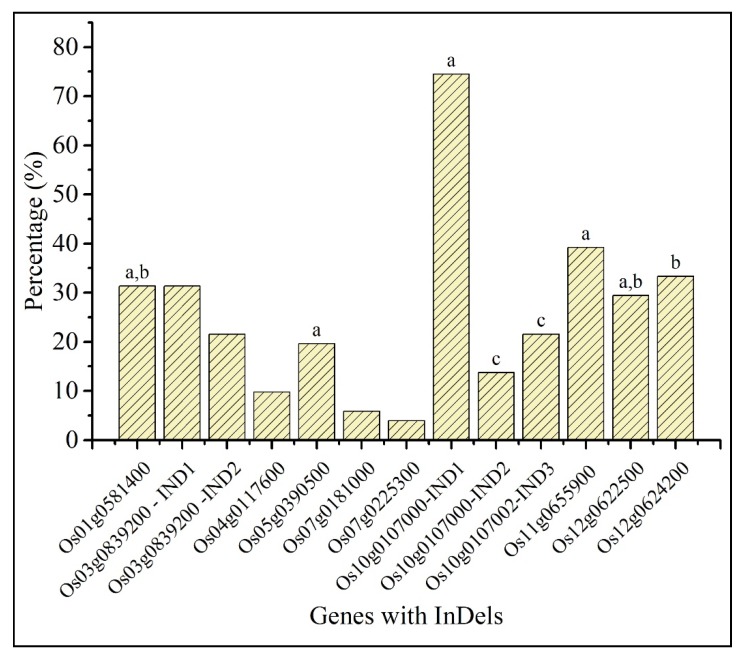
Presence of the InDels in the fifty *indica* rice panel. Inclusion of salt-tolerant donor varieties; Pokkali-IRIS 313-8244, Nona bokra-IRIS 313-7736 and FL478-CX219 are indicated by a, b, and c respectively.

We compared the InDels of the predicted genes in a panel of *indica* rice lines and results revealed that their occurrence varied from approximately 4% to 75%. Of them, the allele of 3bp deletion in *Os10g0107000* was the most abundant InDel while the allele of 9bp insertion in *Os07g0225300* appeared to be a rare allele in the tested population (Figure 4, Supplementary Table S6). We noted that the 12 bp deletion of *Os01g0581400* (GenBank accession number: MK440689) in At 354 was also present in other *indica* varieties such as Nona bokra and Pokkali (Figure 4, Supplementary Figure S1). In At 354, *Os10g0107000* (GenBank accession number: MK492746) possessed a 3 bp deletion, 3 bp insertion and a 9 bp deletion causing a frame shift in amino acid sequence. The same mutations were observed in other salt-tolerant *indica* varieties such as FL478 and Pokkali. *Os11g0655900* had a 6 bp insertion in At 354 (GenBank accession number: MK492750). Interestingly, the same mutation with 6 bp insertion was noted in Pokkali. The 2 bp insertion of *Os12g0622500* in Bg 352 (GenBank accession number: MK492752) was also present in Nona bokra and Pokkali while the 3 bp deletion observed in Bg 352 allele of *Os12g0624200* (GenBank accession number: MK492753) was detected in Nona bokra (Figure 4, Supplementary Figure S1). Accordingly, five genes with InDels found in this study were present in other known salt-tolerant varieties demonstrating evidence for their sequence validation.

### 2.6. Analysis of the Promoter Sequences of the Genes with InDels

We examined the *cis*-acting elements on abiotic stress, within promoter regions of candidate genes detected with Indels, to speculate their association with salinity, comparatively to At 354 and Bg 352. Supplementary Table S7 summarizes the particular *cis*-acting elements found within the 1000 bp 5′ upstream of each of the candidate genes. Nine types of abiotic stress-related *cis*-acting elements were found in this study. They are namely, ABRE, CAAT box, DPBF, GAGA, GBOX, IBOX, ROOT, SEF3, and SEF4 which belong to different transcription factor families involved in abiotic stress-related pathways. The highest number of abiotic stress-related *cis* regulatory elements were found in *Os12g0622500* and the lowest number were found in the *Os04g0117600*. The *Os10g0107000* gene had a comparatively notable difference in terms of the type and the number of *cis*-acting elements. In Bg 352, there were 30 *cis*-acting elements in *Os10g0107000* gene while At 354 had only 24 *cis*-acting elements and the DPBF element was absent in At 354.

### 2.7. PCR-Based InDel Marker for the Detection of Genotypic Polymorphism

**Figure 5:**
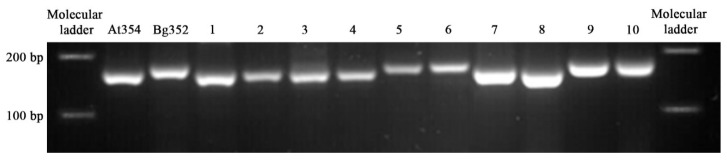
InDel marker for the identification of Polymorphism in *Os01g0581400*. Lane (1). Pokkali-IRIS 313-8244, (2). Kurulutudu-IRIS 313-8925 (3).H6- IRIS 313-9472, (4).Puttu Nellu-IRIS 313-9969, (5).Honderawala-IRIS 313-11382, (6).Herath Banda-IRIS 313-11741, (7 to 10). RILs.3.

Although the accuracy of sequencing is proved, it is still a requirement to confirm the genotypic variations found by *in silico* experiments. Therefore, we selected the longest InDel present among 10 genes, which was the 12 bp deletion in At 354 of *Os01g0581400* allele and designed an InDel marker (PKW) to reveal the polymorphism. The PCR product which was electrophoresed in 3% agarose showed a polymorphic banding pattern matching exactly with the corresponding genotype. Accordingly, At 354, several RILs and International Rice Research Institute (IRRI) germplasm (Pokkali-IRIS 313-8244, Kurulutudu-IRIS 313-8925, H6-IRIS 313-9472 and Puttu Nellu-IRIS 313-9969) were identified as mutated genotype possessing 12 bp deletion in *Os01g0581400* (Figure 5, Supplementary Table S8). The results of this experiment have shown that prediction uncertainty of *in-silico* searchers could be eliminated by combining the task with wet laboratory experiment.

## 3. Discussion

In the present study, the whole genomes of two elite *indica* rice varieties, namely At 354 and Bg 352, were re-sequenced and mapped to both *Oryza sativa* L. cv. Nipponbare reference genome and *Oryza sativa indica* group cultivar R498. As Nipponbare is the current and most comprehensively curated reference genome for the *Oryza sativa*, our analysis was mainly conducted comparatively to Nipponbare while the data were validated using R498 *indica* reference. The near-complete R498 genome is an extra resource for studying genetic variations in rice belonging to *indica* subspecies [45]. Although the majority of reads were mapped to both reference genomes, unmapped read rates of 6% and 29% were observed for At 354 and Bg 352 respectively for Nipponbare and 3% and 27% were observed respectively for R498. The unmapped reads rate of At 354 is comparable with other *indica* rice varieties such as Godawee (8.35%), Swarna (11%) and IR64 (10%) [1,46,47]. Also, Subbaiyan et al. (2012) have observed an average unmapped rate of about 7.5% among 6 *indica* rice inbreds [48]. GC content of At 354 and Bg 352 has been 42.75% and 49.03% respectively, in line with the GC content of monocots that vary within the range of 34% to 49% usually [49]. According to the analysis of chromosome wise variations, a lower number of variants were observed in both varieties with respect to R498 than those of Nipponbare genome. Obviously, it is expected to capture a low number of variants comparatively to R498, because two parents belong to *indica* subspecies. The analysis of chromosome wise variations indicated that IR24, SH527 [50], Godawee [46], Swarna [47] and six elite *indica* rice inbreds [48] contained the highest and the lowest total number of variants on chromosome 1 and 9 respectively as that of At 354 and Bg 352 with respect to Nipponbare. However, the highest and the lowest total number of variants were observed on chromosome 4 and 10 with respect to R498. We calculated the density of occurrence of variants, in order to determine the genomic distribution of SNPs and InDels. The SNP and InDels densities of At 354 and Bg 352 were consistent with other *indica* rice varieties [43,44,45]. As reported by Tenaillon et al. (2001), a greater SNPs rate could be correlated with a higher level of genomic diversity [51]. In the present study, we observed the ratio of transitions to transversions as 2.54 and 2.48 respectively to Nipponbare and R498 indicating more transition SNPs than transversion SNPs showing transition bias. This incidence has been previously reported in rice genomes revealing 2.0 to 2.5 transitions to transversions ratio [46,47,48]. In order to maintain RNA stability and conserve the protein structure, transitional mutations have occurred more frequently than transversions during evolution [52].

In this study, abiotic stress-related genes located within previously identified QTL hotspots were analyzed to identify the variants in At 354 and Bg 352. Altogether, we used 14 QTL hotspots flanked less than 1 Mb intervals explaining 12.5–46.7% of phenotypic variation in salinity-related traits [35]. We could find 106 abiotic stress-related genes associated with these QTL hotspots. According to a frequency distribution analysis of genome-wide variations conducted by Jiang et al. (2017) [53], 10 highest SNPs and InDels rich regions were identified in the rice genome. Of them, three regions, chromosome 2 (33–35 Mb), 5 (19–22 Mb) and 6 (10–22 Mb) were exactly matched with the intervals of QTL hotspot 2, 4 and 8 of At 354 × Bg 352, respectively with reference to both Nipponbare and R498. This observation gives evidence to justify the polymorphic nature of the respective QTL regions, indicating the possible existence of allelic variations. Also, we detected the highest number of stress-related genes (19) on the QTL hotspot 2 indicating its potential contribution for salt responsive phenotypic variation. QTL hotspots were previously detected under phytotron conditions of the International Rice Research Institute, Philippines where all possible salinity-related QTLs might not have been expressed [35]. Therefore, in addition to the abiotic stress-related genes within the QTL hotspots, we considered 27 other abiotic stress-related genes which were involved in salt-tolerant pathways. Hence, altogether 133 genes were analyzed and polymorphic variants between At 354 and Bg 352 were observed to identify potential candidate genes. As exons are significantly important due to their function in presenting mRNA and coding the proteins, here we focused mainly on the alternative variants in exons between At 354 and Bg 352.

The SNPs are single-point mutations observed in the genomic DNA of organisms. Some of the SNPs cause the amino acid substitution in the corresponding amino acid sequence (missense mutations) of the genes while others are not (silent mutations) [54]. The missense mutations affect the protein function indirectly through effects on protein folding, stability, flexibility, and aggregation. Modification of the protein to be more flexible or rigid, compared to the respective native structure affects the protein function adversely. If a missense mutation occurred in an active site of a protein structure, it could possibly alter the biological or biochemical reactions and change the kinetics of the reaction and affect the normal protein function [54,55]. There are a number of studies that have shown the functional consequences of SNPs. Wang et al. (1997) [56] have shown that a missense mutation (*adg2-1*) in the ADPG Pyrophosphorylase large subunit gene either affects the stability of the ADGase large subunit protein or its assembly into holoenzyme in *Arabidopsis thaliana*. The *S1-24* mutant in a highly conserved zinc finger domain of *OsCESA7* gene in rice is due to a missense mutation, causing brittle culms, dwarfism and partial sterility. The influence of this mutation is predicted to be in affecting the interactions between different CESA subunits and *OsCESA7* [57]. Tang et al. (2018) [58] have shown that a missense mutation in a plastid ribosomal protein (*RPS4*) in Chinese cabbage has impaired the rRNA processing and affected the ribosomal function. This information indicates that missense mutations occurred due to SNP variations in the gene sequences play an important role in affecting the gene functions in plants. Thus, *in silico* information on SNP variants reported in the present study, could possibly play an important role in regulating the functions of the genes under abiotic stress condition.

The candidate genes with InDels identified in the present study were compared with past research studies in order to speculate their function in relation to stress tolerance. Due to the fact that InDels of *Os01g0307500* and *Os04g0423400* did not appeared with reference to the R498, we did not consider them as true variants.

The gene, *Os01g0581400* identified in the present study, also was reported by Chen et al. (2010) [36] indicating that it contains a juxtamembrane (JM) domain which regulates the proper function of receptor-like kinases (RLKs) by autophosphorylation. The RLKs play an important role in plant responses such as development, hormone perception, defense and response to pathogens [59,60]. *Os01g0581400* had a 12 bp deletion in At 354 (GenBank accession number: MK440689) which truncated the sequence to 761 amino acids while Bg 352 (GenBank accession number: MK440690) showed coding of full sequence with 765 amino acids. This gene was found from QTL hotspot 1 and At 354 had been the respective allele donor for salt tolerance as shown by additive effect (Table 3). GO analysis also indicated that it functions as a protein kinase (GO:0004672) and its involvement in protein phosphorylation (GO:0006468) (Supplementary Table S5). It is interesting to note that At 354 allele which had 12 bp deletion leading to altered amino acid sequence was also present in Nona bokra and Pokkali which are popular salinity tolerant varieties (Supplementary Figure S1). Moreover, we could reveal the two types of alleles detected, in *in silico* analysis using a novel InDel marker confirming their physical presence in diverse varieties (Figure 5). Although the presence of two types of alleles of *Os01g0581400* was proved in the RIL population and other diverse germplasm, we could not interpret the haplotype contribution to salt responsiveness due to genetic complexity of the trait, because none of the salt-tolerant donors has all the desirable alleles for tolerance mechanisms while a salt-susceptible line may also contain few desirable alleles, affecting for unpredictable cumulative effect. The same perception was supported by Islam et al. (2019) [61], who reported the same gene, *Os01g0581400* as a salt responsive candidate gene indicating that possible activation of protein kinase domain-containing protein (*LOC_Os01g39970*) of the particular gene under salt stress by QTL-meta-analysis, a precise estimation technique. However, they were also unable to demonstrate the association between the genes with meta-QTL linked makers and salt tolerance due to complexity of the trait. Therefore, we suggest that the InDel marker developed from the mutation of *Os01g0581400* would be useful to develop near-isogenic lines nullifying the complexity caused by other genes, to investigate the allele contribution for salt tolerance.

The *Os01g0583100,* potentially being another candidate gene located on the same QTL hotspot, possessed one missense mutation (Supplementary Table S4). It was reported as protein phosphatase 2C (*PP2C6)* family member of rice. The *Os01g0583100* is regulated by ABA via ABA-responsive elements located on its promoter (GO:0048364, GO:0004722) (Supplementary Table S5) [62]. Yoshida et al. (2010) [63] have reported the function of PP2C genes in relation to water stress (GO:0009414) and drought conditions while Li et al. (2015) [64] have reported their importance in controlling root architecture and drought tolerance.

The *Os01g0591000* (*OsALDH2C4*) and *Os05g0455500* (*OsALDH18B1*), possessing missense mutations, located on QTL hotspot 1 and 7 respectively (Supplementary Table S4), were found belonging to rice aldehyde dehydrogenase (ALDH) protein superfamily [65]. Kotchoni et al. (2010) [65] have reported that *OsALDH18B1* which is unique for rice, encodes an enzyme for proline synthesis (*P5CS*) (GO:0006561, GO:0004029, GO:0043878) and is important for salt stress adaptation and tolerance. Moreover, the ALDHs are capable of detoxifying the reactive aldehyde molecules which are produced under different abiotic stress conditions and maintain the redox balance in the cells [65,66].

The candidate gene *Os02g0766700* (*OsbZIP23*) located on QTL hotspot 2, is a member of basic leucine zipper (bZIP) transcription factor family in rice, and it contains two missense variants in Bg 352 variety when compared to the At 354. Several gene expression studies have reported its sensitivity to drought, salt and osmotic stress responses [37,67]. Moreover, Xiang et al. (2008) have observed that *OsbZIP23* is highly expressed in leaf tissues and its overexpression may enhance salt tolerance [38]. GO analysis also indicated its involvement in response to water deprivation (GO:0009414) and salt stress (GO:0009651).

The *Os02g0782500* screened with a missense mutation, was also identified previously as an abiotic stress-responsive gene in rice and the function was categorized under small heat stress protein (sHSP) class III by Yi et al. (2013) [68]. Also, Waters et al. (2008) [69] have reported that sHSPs are expressed in other plant response stresses such as drought, salinity, UV, osmotic and oxidative stresses in addition to heat and cold responses (GO:0009408, GO:0009644, GO:0009651, GO:0042542) (Supplementary Table S5). Therefore, the polymorphism between two alleles needs to be further characterized with regards to the function under salinity.

In our study, the salinity susceptible variety, Bg 352 was found with a 3bp insertion, 3bp deletion and three missense variants in *Os03g0839200* (GenBank accession number: MK440692) that shifted the frame of the amino acid from 490 to 516. In accordance with the additive effect, this mutation indicates a possible contribution to salt susceptibility. Neerja et al. (2018) [70] have conducted a research on the transporter genes and found that the *Os03g0839200* has been associated with multidrug and toxic compound extrusion (MATE) efflux family protein (GO:0006855, GO:0015238, GO:0015297)*,* which is an integral component of the membrane involving in salt toxic ion extrusion.

The gene, *Os04g0117600* was found possessing a 3 bp insertion in Bg 352 (GenBank accession number: MK492739) and the mutation caused a frame shift in amino acid sequence starting from 310 position and terminated with 690 amino acids. At 354 predicted the full sequence with 689 amino acids (GenBank accession number: MK492738). It was noted that the gene was located in the telomeric region of chromosome 4. According to the GO analysis, *Os04g0117600* could be involved in tRNA dihydrouridine synthesis (GO:0002943), metal ion binding (GO:0046872) and oxidation reduction processes (GO:0055114).

An InDel variation was found in *Os05g0390500* in QTL hotspot 7 located at 19.8–20.5 Mb of chromosome 5. GO analysis indicated that *Os05g0390500* is responsive to salt stress (GO:0009651) (Supplementary Table S5). *Os06g0318500* gene found with four SNP variations, is one of the five Na^+^/H^+^ exchanger (NHX) genes present in rice (GO:0009651, GO:0015385, GO:0015386) and several studies have shown that *NHX* genes are capable of regulating the Na^+^ and/or K^+^ uptake under high salinity conditions faced by plants [41,42,71]. Yang et al. (2016) [72] have reported that *Os07g0181000*, which contained a 6 bp insertion mutation in At 354 allele (GenBank accession number: MK492744), is a photosynthesis-related gene. However, the relevance of the gene to salinity needs to be further investigated.

The *Os10g0107000* on QTL hotspot 11 was found with 3 InDels including a 3 bp insertion, 9 bp and 3 bp deletions in At 354 (GenBank accession number: MK492746) and the same mutations were also observed in other salt-tolerant *indica* varieties, FL478 and Pokkali. *Os10g0107000* which was identified as a class III peroxidase family gene (GO:0004601, GO:0006979) has been upregulated in response to cadmium stress in rice [43]. In addition, Wang et al. (2015) [73] have observed that class III peroxidase genes are differentially expressed in response to abiotic stress in maize and play a significant role in roots.

Another two genes, *Os11g0655900* (*OsGRX23)* and *Os11g0656000* (*OsGRX24)* belonged to CC-type Glutaredoxin (GRX) family contained an InDel and missense mutations respectively. It was reported that GRXs regulate and participate in the redox-dependent signaling pathways (GO:0045454, GO:0009055, GO:0022900) and provide protection to plants over oxidative stress while being involved in several metabolic pathways [74,75]. Moreover, Garg et al. (2010) [74] have shown that *Os11g0655900* is differentially expressed in rice seedlings under different abiotic stress conditions. The *Os11g0655900* gene possessed a 6 bp insertion in At 354 (GenBank accession number: MK492750) while Bg 352 (GenBank accession number: MK492751) closely aligned with Nipponbare and R498. We noted that the allele contribution for salt tolerance was by At 354 although distortion was occurred by the extension of two additional amino acids. However, reference genomes- (Nipponbare and R498)-based alignments do not indicate which could be the distorted allele, whether At 354 or the reference genomes. Sometimes the At 354 allele could encode the correct version of the amino acid sequence because it gives two additional amino acids. Also, we observed that Pokkali contained the same 6 bp insertion in the particular location indicating that prevalence of the same allele in another salt-tolerant donor variety.

We observed that *Os12g0622500* gene in Bg 352 (GenBank accession number: MK492752) had a 2 bp insertion truncating the sequence to 323 amino acids due to early gain of stop codon while At 354 had the full sequence length with 487 amino acids. We observed that the same mutation was present in Nona bokra giving evidence for the prevalence in another salt-tolerant donor variety. The *Os12g0624200* belonging to the Ca^2+^/cation antiporter superfamily (GO:0055085) was detected with a 3 bp deletion in Bg 352 (GenBank accession number: MK492753) that shifted the amino acid frame starting from 30th amino acid position with reference to the Nipponbare and R498 genomes. Also, the allele of Bg 352 truncated the sequence to 586 amino acids while At 354 encoded the full sequence with 587 amino acids. Furthermore, studies have shown that *Os12g0624200* is significantly upregulated in response to salinity and dehydration conditions imposed on rice suggesting its involvement in stress tolerance [76,77].

Not only the coding sequences, but also the 5′ upstream regions including the *cis*-acting elements of promoter sequences usually affect the expression of the genes. Therefore, analyzing variations of the *cis*-acting elements gives an insight into the understanding of functional variations of genes. Hence, we analyzed the *cis*-acting elements of the 10 candidate genes with InDels to examine their involvement in stress-related pathways and also to speculate possible causal factors in addition to the InDel variations (Supplementary Table S7). As a whole, the analysis of *cis*-acting elements in the promoter regions of the candidate genes, ABRE, CAAT box, DPBF, GAGA, GBOX, IBOX, ROOT, SEF3, and SEF4 were present approximately an equal number in all genes indicating their involvement in stress-related mechanisms as reported in several studies [78,79,80,81,82,83,84].

Recent research advances have shown that environmental stresses, do not only imbalance the ionic and osmotic homeostasis in plants but also weaken photosynthesis, redox reactions, and cellular energy depletion. Therefore, plants harbor a broader, overlapping set of genes that are involved in both biotic and abiotic stress responses and developmental processes, increasing the evidence that plant signaling does not operate as independent or parallel pathways [85]. Golldack et al. (2014) [86] have proposed a model on cross-talk of ABA, gibberellic acid and jasmonate signaling plant responses to abiotic stressors such as drought and salt, linking with other pathways leading to ROS detoxification, lipid signaling and structural adaptation of membranes. Thus, the ABA-related candidate genes found in this study (*Os01g0583100 and Os02g0766700*) could be involved in different inter-connected networks and pathways to function against salinity stress. Similarly, bZIPs, RLKs, PP2Cs, and other candidate genes found in this study would be involved in linking abiotic stresses such as heat, cold, osmotic, oxidative and salinity stress by mediating signaling cross-talks [14,85,87,88].

## 4. Materials and Methods

### 4.1. Plant Material and DNA Extraction

At 354 and Bg 352 varieties were selected for whole genome re-sequencing in this study. The rice seeds were grown under controlled conditions and the genomic DNA was extracted from leaf tissues of 2-week-old seedlings of At 354 and Bg 352 using the CTAB method [89].

### 4.2. Rice Whole Genome Re-Sequencing and Variant Calling

High throughput whole genome re-sequencing was performed using Illumina’s paired-end sequencing technology on the Hiseq 2000 platform for two rice varieties. The paired-end libraries were constructed for Bg 352 and At 354 according to the manufacturer’s protocol (Illumina Inc., Hayward, CA 94545, USA). After the clonal cluster generation, the DNA was sequenced by Illumina’s sequencing by synthesis (SBS) technology. The sequencing data were converted into raw data with 101 bp size reads and obtained BAM and fastq files for further analysis. After the raw reads were gone through the quality control process, the quality-filtered reads were mapped to two reference genomes, *Oryza sativa japonica* group cultivar Nipponbare IRGSP-1.0 (GenBank Assembly Accession: GCA_001433935.1) and *Oryza sativa indica* group cultivar Shuhui498 (R498) (GenBank Assembly Accession: GCA_002151415.1) using Burrows Wheeler Alignment (BWA) program [90] with default parameters. The duplicates in the aligned reads were removed and the alignment results were merged to generate indexed BAM files. Basic statistics including GC%, read depth, coverage and Q20/Q30 were calculated using the alignment results. The mapped reads were used to detect SNPs and InDels. After removing duplicates with Sambamba and identifying variants with SAMTools, information on each variant was gathered and classified by chromosomes. The variants were further filtered using parameters i.e., variant quality score ≥100 and zygosity (homozygous). Circos software was used to visualize the frequency distribution of the SNPs and InDels on 12 rice chromosomes of At 354 and Bg 352 with respect to Nipponbare genome and R498 genome.

### 4.3. Variation Analysis on Abiotic Stress-Related Genes and Prediction of Candidate Genes for Salinity

The QTL hotspot regions which were previously identified by QTL mapping of At 354 × Bg 352 were queried for prospective abiotic stress-related genes in the regions [35]. In addition, known salt tolerance-related genes were also selected. Gramene and NCBI GenBank Database [91,92] were used for identifying the abiotic stress-related genes. IRGSP-1.0 annotations were used in identifying the locations of the genes within QTLs. Respective coordinates of the R498 were obtained by matching the DNA sequences of IRGSP-annotated genes with R498. The SNP and InDel variants of the genes in the QTL regions of At 354 and Bg 352 genomes were classified according to their locations such as exons, introns, 5′ and 3′ untranslated regions (UTR) with respect to the Nipponbare. The SNPs and InDels which were polymorphic between At 354 and Bg 352 in the exons were further examined in the R498 reference. The amino acid changes due to SNPs were observed based on the Short Genetic Variations database (dbSNP) of NCBI [93]. The open reading frames (ORF) for the coding sequences (CDS) of the selected genes (with InDels) were predicted by NCBI ORF finder [94].

### 4.4. Comparative Analysis of InDels in Predicted Candidate Genes with indica Rice Lines

The nucleotide sequences of predicted genes in a panel of 50 rice cultivars including Sri Lankan rice varieties and popular salt-tolerant donor varieties, FL 478 (CX219), Nona bokra (IRIS 313-7736) and Pokkali (IRIS 313-8244) were retrieved from Rice SNP-Seek Database [95]. The concordance of InDel variations was examined with *indica* rice panel.

### 4.5. Analysis of the Promoter Sequences of the Genes with InDels

The 1000 bp 5′ upstream region of the selected candidate genes were retrieved for At 354 and Bg 352 varieties as the promoter sequences. The plant *cis*-acting regulatory DNA elements of each gene were obtained from the NEW PLACE database version 30.0 [96]. Out of the total *cis*-acting elements, the abiotic stress-related *cis*-acting elements were filtered using the already available literature [97].

### 4.6. GO Analysis

The candidate gene sequences of At 354 and Bg 352 were annotated with Blast2GO software using the blastn algorithm and the Cloud Blast database in order to identify the molecular function, biological process and the cellular components [98].

### 4.7. Data Availability

The gene sequence data for this study can be found at GenBank Repository. (https://www.ncbi.nlm.nih.gov/genbank/).

The VCF files for At 354 and Bg 352 with reference to Nipponbare and R498 genomes has been archived under the following accessions of EVA. (https://www.ebi.ac.uk/ena/data/view/PRJEB35319).

Project: PRJEB35319.

Analyses: ERZ1143791, ERZ1143792, ERZ1143793, ERZ1143794, ERZ1143795, ERZ1143796, ERZ1143797, ERZ1143798, ERZ1143799, ERZ1143800.

## 5. Conclusions

In this study, we re-sequenced two elite *indica* rice varieties—At 354 (salt-tolerant) and Bg 352 (salt susceptible)—with reference to *japonica* cultivar Nipponbare and *indica* cultivar R498 and detected high genetic variations through SNPs and InDels between two parents, particularly in their chromosomes and QTL regions. We identified a narrow deviation in QTL locations between Nipponbare and R498 references, ranging from 0.1 Mb to 3 Mb. In total, 106 abiotic stress-related genes were identified in QTL regions, most of which had polymorphic nucleotide variants between two parents. Of them, 34 genes were identified for the presence of polymorphic SNPs and InDels between parents with respect to Nipponbare, but only 32 variants were confirmed with the reference material of R498. Altogether 10 genes that contained InDels leading to altered amino acid sequences were identified and their mutated sequences were able to be validated due to their presence in other *indica* varieties. Further studies need to be focused on the functional characterization of particular alleles by expression studies under different salinity levels and exposure times in order to elucidate the contribution of mutations on salt tolerance. The different haplotypes revealed in this study would be useful for genetic improvement of rice through haplotype-based molecular breeding.

## Figures and Tables

**Table 1:** Summary of sequencing statistics.

Reference	Ref Length		Mapped Sites	Total Reads	Mapped Reads	Mapped Bases	Mean Depth	GC%	Ts/Tv
**Nipponbare**	374,304,577	At 354	349,124,521 (93.27%)	114,142,434	108,034,211 (94.65%)	10,446,593,443	27.91	42.75	2.54
		Bg 352	348,205,846 (93.03%)	135,985,268	96,223,079 (70.76%)	9,333,912,611	24.94	49.03	
**R498**	390,983,850	At 354	374,732,599 (95.84%)	114,127,820	110,684,704 (96.98%)	10,689,439,220	27.34	42.75	2.48
		Bg 352	373,811,968 (95.61%)	135,973,740	98,099,869 (72.15%)	9,548,473,921	24.42	49.03	

**Table 2:** Occurrence and density of SNPs and InDels within the 12 chromosomes in At 354 and Bg 352 genomes after quality filtering.

**At 354**										
**Ch**	**No of SNPs**		**Density (SNPs/100 kb)**		**No of InDels**		**Density (InDels /100kb)**		**Total No of Variants**	
	**Nipponbare**	**R498**	**Nipponbare**	**R498**	**Nipponbare**	**R498**	**Nipponbare**	**R498**	**Nipponbare**	**R498**
1	285,179	118,433	659.1	267.0	31,896	7703	73.7	17.4	317,075	126,136
2	242,403	72,315	674.5	191.5	26,628	5104	74.1	13.5	269,031	77,419
3	230,918	76,883	634.1	193.7	25,899	5133	71.1	12.9	256,817	82,016
4	198,076	121,443	557.9	338.8	19,779	6776	55.7	18.9	217,855	128,219
5	167,936	75,130	560.6	240.5	18,590	4704	62.1	15.1	186,526	79,834
6	217,319	89,233	695.4	274.9	22,179	5432	71	16.7	239,498	94,665
7	207,603	79,157	699.1	261.4	20,678	5046	69.6	16.7	228,281	84,203
8	186,119	91,487	654.4	305.4	19,016	11,184	66.9	37.3	205,135	102,671
9	166,630	78,222	724.1	315.9	16,561	6223	72	25.1	183,191	84,445
10	180,213	52,393	776.5	204.8	17,107	4854	73.7	19.0	197,320	57,247
11	218,524	94,898	753	298.6	20,369	8158	70.2	25.7	238,893	103,056
12	177,449	95,189	644.5	357.8	16,929	7626	61.5	28.7	194,378	102,815
**Bg 352**										
**Ch**	**No of SNPs**		**Density (SNPs/100kb)**		**No of InDels**		**Density (InDels /100kb)**		**Total No of Variants**	
	**Nipponbare**	**R498**	**Nipponbare**	**R498**	**Nipponbare**	**R498**	**Nipponbare**	**R498**	**Nipponbare**	**R498**
1	282,510	112,753	652.9	254.2	31,008	7567	71.7	17.1	313,518	120,320
2	234,214	87,750	651.7	232.4	25,297	5958	70.4	15.8	259,511	93,708
3	240,771	82,763	661.2	208.5	26,549	5411	72.9	13.6	267,320	88,174
4	214,856	151,002	605.2	421.2	20,144	7562	56.7	21.1	235,000	158,564
5	160,435	77,146	535.5	247.0	17,570	4690	58.6	15.0	178,005	81,836
6	211,331	65,387	676.3	201.4	21,022	4176	67.3	12.9	232,353	69,563
7	215,710	49,767	726.4	164.4	21,225	3449	71.5	11.4	236,935	53,216
8	180,107	75,759	633.2	252.9	17,993	8474	63.3	28.3	198,100	84,233
9	159,509	74,401	693.1	300.5	15,603	6456	67.8	26.1	175,112	80,857
10	180,638	49,286	778.4	192.7	16,604	3137	71.5	12.3	197,242	52,423
11	215,175	108,091	741.4	340.1	19,436	6214	67	19.6	234,611	114,305
12	181,988	104,139	661.0	391.5	16,774	5774	60.9	21.7	198,762	109,913

**Table 3:** Candidate genes identified based on polymorphic InDels in exons regions of two parents.

			Nipponbare				R498				
Gene	QTL Hotspots and Additive Effect ^a^	Variation Type	Location	Reference	At 354	Bg 352	Location	Reference	At 354	Bg 352	Amino acid Position
*Os01g0581400*	01, At 354	SNP	22539348	A	G	A	23425343	G	G	A	I 20 M
		SNP	22539497	A	G	A	23425492	G	G	A	N 70 S
		Indel	22540408	ACTGCGGCGGCGGC	AC	ACTGCGGCGGCGGC	23426414	AC	AC	ACTGCGGCGGCGGC	frame shift
*Os03g0839200*	03, At 354	SNP	35266328	A	A	G	38332616	A	A	G	D 20 G
		SNP	35266337	T	T	C	38332625	T	T	C	V 23 A
		SNP	35267146	G	G	A	38333434	G	G	A	D 293 N
		Indel	38335687	CGAAG	CGAAG	CG	38335687	CGAAG	CGAAG	CG	frame shift
		Indel	35269310	CC	CC	CCATC	38335755	CC	CC	CCATC	frame shift
*Os04g0117600*	06, Bg 352	SNP	1047005	C	C	T	1342824	T	C	T	P 16 L
		SNP	1047150	G	A	G	1342968	G	A	G	G 64 D
		SNP	1048077	C	C	T	1343864	T	C	T	T 134 M
		SNP	1048092	A	A	G	1343879	G	A	G	N 139 S
		Indel	1051506	AA	AA	AAGTA	1346152	AAGTA	AA	AAGTA	frame shift
		SNP	1053033	G	C	G	1347696	G	C	G	S 585 T
*Os05g0390500*	07, At 354	Indel	18933030	AGG	AGGGG	AGG	20113492	AGGGG	AGGGG	AGGGG	frame shift
		SNP	18933023	T	T	G	20113527	G	T	G	S 529 A
*Os07g0181000*	09, Bg 352	Indel	4266957	CGCCAC	CGCCACAGCCAC	CGCCAC	4219095	CGCCAC	CGCCAC	CGCCAC	frame shift
Os07g0225300 *		Indel	6968059	TGGCGGCG	TGGCGGCGTCGGCGGCG	TGGCGGCG	6890507	TGGCGGCG	TGGCGGCGTCGGCGGCG	TGGCGGCG	frame shift
		SNP	6968496	G	G	A	6890944	A	G	A	G 147 S
		SNP	6968832	A	T	A	6891280	A	T	A	M 259 L
		SNP	6968847	C	G	C	6891295	C	G	C	P 3264 A
*Os10g0107000*	11, Bg 352	Indel	482854	GGTCGTCG	GGTCGTCGTCG	GGTCGTCG	686457	GGTCGTCG	GGTCGTCGTCG	GGTCGTCG	frame shift
		SNP	483138	C	C	T	686741	T	C	T	S 179 N
		SNP	489170	C	G	G	686974	G	G	G	E 101 D
		Indel	489349	CGCCGCCGGAGCCG	CGCCG	CGCCGCCGGAGCCG	687153	CGCCGCCGGAGCCG	CGCCG	CGCCGCCGGAGCCG	frame shift
		Indel	489388	CTGATGA	CTGA	CTGATGA	687195	CTGATGA	CTGA	CTGATGA	frame shift
*Os11g0655900*	13, At 354	Indel	26270259	CG	CGCCGGAG	CG	29205256	CG	CGCCGGAG	CG	frame shift
		SNP	26270278	C	C	G	29205275	G	G	G	L 71 V
*Os12g0622500*	14, Bg 352	SNP	26579061	A	A	G	25614979	G	A	G	H 15 R
		Indel	26579981	ATT	ATT	ATTTT	25615899	ATTTT	ATT	ATTTT	frame shift
*Os12g0624200*	14, Bg 352	Indel	26669266	CCGTCGTCGTCGTCGTC	CCGTCGTCGTCGTCGTC	CCGTCGTCGTCGTC	25694767	CCGTCGTCGTCGTC	CCGTCGTCGTCGTCGTC	CCGTCGTCGTCGTC	frame shift

* Not within QTL hotspots ^a^ Allele donor was obtained from Additive effect of Gimhani et al. (2016) [35].

## Supplementary Materials

The following are available online at https://www.mdpi.com/2223-7747/9/2/233/s1. Supplementary Table S1. Supporting references for QTL hotspots co-located in the same chromosomal regions reported in other breeding studies. Supplementary Table S2. Variants identified in abiotic stress-related genes with reference to Nipponbare genome. Supplementary Table S3. Abiotic stress-related *cis* acting elements revealed by the analysis of the promoter sequences of the genes with InDels. Supplementary Table S4. Candidate genes identified based on polymorphic SNPs in exons regions of two parents. Supplementary Table S5. GO analysis of the selected candidate genes. Supplementary Table S6. Presence of InDels in the selected rice panels. Supplementary Figure S1. *InDels* detected in the candidate genes and their sequence validation. A. *Os01g0581400* B. *Os10g0107000* C. *Os11g0655900* D. *Os12g0622500* E. *Os12g0624200*. Supplementary Table S7 Abiotic stress-related Cis acting elements revealed by the analysis of the promoter sequences of the genes with InDels. Supplementary Table S8 InDel marker information.
